# The relationship between social support and depression among HIV-positive men who have sex with men in China: the chain mediating role of psychological flexibility and hope

**DOI:** 10.3389/fpubh.2023.1271915

**Published:** 2023-11-08

**Authors:** Run Wang, Fang Zheng, Guiying Cao, Lloyd A. Goldsamt, Yan Shen, Ci Zhang, Mengyao Yi, Wenwen Peng, Xianhong Li

**Affiliations:** ^1^Xiangya School of Nursing, Central South University, Changsha, Hunan, China; ^2^Department of AIDS, The First Hospital of Changsha, Changsha, Hunan, China; ^3^Rory Meyers College of Nursing, New York University, New York, NY, United States

**Keywords:** HIV, men who have sex with men, social support, psychological flexibility, hope, depression

## Abstract

**Introduction:**

HIV and mental health problems are a global syndemic. One key issue is that the significant mental health problems among people vulnerable to acquiring or living with HIV have not been fully addressed. Access to social support has been one of the biggest challenges for HIV-positive men who have sex with men (HIV+ MSM). Lower social support has been linked to more severe depression symptoms. However, the mechanisms underlying the association between social support and depression in HIV+ MSM are unclear. Two possible mediators include hope and psychological flexibility. This study aimed to examine the relationship between social support and depression in HIV+ MSM and to explore the single mediating effects of hope and psychological flexibility and the chain mediating effect of these two variables on this relationship.

**Methods:**

A convenience sample was used to recruit participants from the designated HIV/AIDS hospital in Changsha city, Hunan Province of China. A total of 290 HIV+ MSM completed questionnaires.

**Results:**

Our findings showed that hope mediated the relationship between social support and depression in HIV+ MSM. Furthermore, the chain mediation model confirmed a direct negative association between social support and depression, but this relationship was largely mediated by the chain effects of hope and psychological flexibility.

**Conclusions:**

Integrating hope and psychological flexibility into interventions may provide better mental health support for HIV+ MSM and improve their wellbeing and quality of life.

## Introduction

Globally, there has been a rapid increase in HIV rates in men who have sex with men (MSM) in the last decade (1). The risk of HIV infection for MSM was 26 times higher than that of other adult males (2). China has a similar HIV epidemic trend. Among people newly diagnosed with HIV in China, the proportion of MSM increased from 1.5% in v2006 to 23.0% in 2019, making MSM the fastest-growing sub-group among those infected (3). Under the traditional Chinese cultural background of “filial piety,” homosexuality was morally unacceptable. Structural and psychological forms of stigma toward MSM, including social exclusion and marginalization, create cycles of co-occurring HIV and mental health disorders (4). Thus, Chinese HIV-positive MSM (HIV+ MSM) are even more vulnerable to mental health problems compared with other populations. The anxiety and depression rates among Chinese HIV+ MSM were 38.7 and 50.5% (5), respectively, while they were 25% (6) and 31% (7) among people living with HIV/AIDS (PLWHA) and 2.1 and 5.0% among the Chinese general population, respectively (8). Moreover, mental health problems among HIV+ MSM adversely impact HIV treatment outcomes and lower their quality of life. Depressive symptoms have consistently been found to predict HIV symptoms, drug resistance, and even mortality (4, 9–11). Evidence has also confirmed that poor mental health negatively affects treatment outcomes, including lowering CD4 counts and quality of life (9, 11). Given the highly stigmatized cultural background toward homosexuality, improving the mental health of Chinese HIV+ MSM remains challenging. Outcomes are influenced by patients' coping resources (e.g., social support) and coping responses (e.g., active and avoidant) according to the Stress-Processing Model (12). Among those influencing factors, access to social support has been widely proven to be one of the biggest challenges for PLWHA (13, 14). In addition, according to the Stress-Processing Model, the process of enhancing active coping responses (e.g., psychological flexibility and hope) is necessary for mental health, which may influence depressive symptoms (12). However, there is no research on the pathways between social support and depression among HIV+ MSM. Therefore, this study aims to explore the mediating mechanism of the relationship between social support and depression among HIV+ MSM by understanding the mediating factors to provide recommendations for improving the mental health of HIV+ MSM.

As the main resource of social support, family support could help HIV-infected individuals develop spiritual strength, establish life goals, reshape the meaning of life, and increase treatment adherence, thus enhancing the level of hope (15). Unfortunately, access to social support for HIV+ MSM is not encouraging. Evidence has shown that the availability of social support for Chinese HIV+ MSM was 20% lower than that of other adult males (13) due to HIV-related stigma. Furthermore, due to the fear of being stigmatized, HIV+ MSM were reluctant to access those available social resources (16, 17). Among HIV+ MSM, social support has been associated with reduced hopelessness, anxiety, and depressive symptoms. Evidence suggests that strong social support may reduce the risk of depression by up to 50% among HIV+ MSM (13). Moreover, African Americans living with HIV clearly expressed a desire for emotional and instrumental support and emphasized support as a mechanism to resist the negative effects of HIV-related stigma (18). However, Parcesepe et al. (19) suggested that there was no direct association between social support and psychological distress among PLWHA, which was inconsistent with previous studies. Social support may be indirectly associated with depression through other mediators. However, the evidence illustrating how social support may be positively linked to mental and physical health outcomes is less conclusive, although essential when designing effective interventions for HIV+ MSM (20, 21). Therefore, it is critical to explore potential pathways of social support and depression in HIV+ MSM.

Psychological flexibility might be a potential mediator in explaining how lower social support may lead to higher depression. According to the Stress-Processing Model, psychological flexibility plays an important role in improving mental health, which could not only directly reduce depression but also alleviate negative emotional experiences (22, 23). Psychological flexibility refers to the capacity of individuals to use psychological resources flexibly to adapt to different psychological needs or change their own points of view according to the environment (24). However, due to the life-long experience of sexual minority stress, HIV+ MSM demonstrate a lower level of psychological flexibility, leading them to keep their HIV infection a secret and consistently worry about being stigmatized (25, 26). Meanwhile, previous studies have confirmed that greater psychological flexibility was associated with optimal mental health (25–27). For example, a cross-sectional study among HIV+ MSM in the United States showed that psychological flexibility was an important predictor of mental health disorders (26), and lower psychological flexibility was associated with higher levels of depression (26). Despite growing evidence of the positive effects of psychological flexibility, the role of psychological flexibility in the relationship between social support and depression in people living with HIV remains unclear. Based on the existing evidence (28–30), this study hypothesized that psychological flexibility might become a mediator for HIV+ MSM's ability to access social support and reduce their depression.

Hope might be an additional potential mediator explaining the pathway from social support to depression. Social support is strongly associated with hope. As an intrinsic resource, social support, especially family support, could also strengthen hope. A study among 160 HIV-infected patients in Nepal showed that social support had a positive influence on hope, with higher social support associated with higher levels of hope (31). Evidence also indicates that hope is an important factor that affects the mental health of PLWHA (32–34). Liu et al. (34) conducted a study among 206 PLWHA and found that higher levels of hope could reduce depression symptoms, help PLWHA accept their HIV infection, and rationalize their sexual behavior. Individuals with higher levels of hope may possess a greater ability to cope with difficulties (34). Although no research has yet found a mediating role for hope between social support and depression among HIV+ MSM, it is a mediator between social support and quality of life in PLWHA (31), suggesting that hope may alleviate the negative impact of inadequate social support on quality of life. Given the association between social support and depression (31, 34), it was assumed that hope could also serve as a mediator between social support and depression among HIV+ MSM (35).

Moreover, the literature shows that hope is also associated with psychological flexibility (12). According to the Stress-Processing Model, hope as a positive psychological coping mechanism should be interrelated with depression (36), but this relationship could be mediated by psychological flexibility, as indicated by a study conducted among the general population in France during the COVID-19 lockdown period (35). Arslan et al. (37) also found that psychological inflexibility mediated the relationship between optimism-pessimism and depression among Turkish young adults, suggesting that hope and psychological flexibility may work together to alleviate depressive symptoms. However, few studies have neither examined the mediating roles of psychological flexibility and hope between social support and depression among HIV+ MSM, nor the combined impact of the two factors on depression, and the underlying mechanism of these associating variables remains unclear.

**Figure 1:**
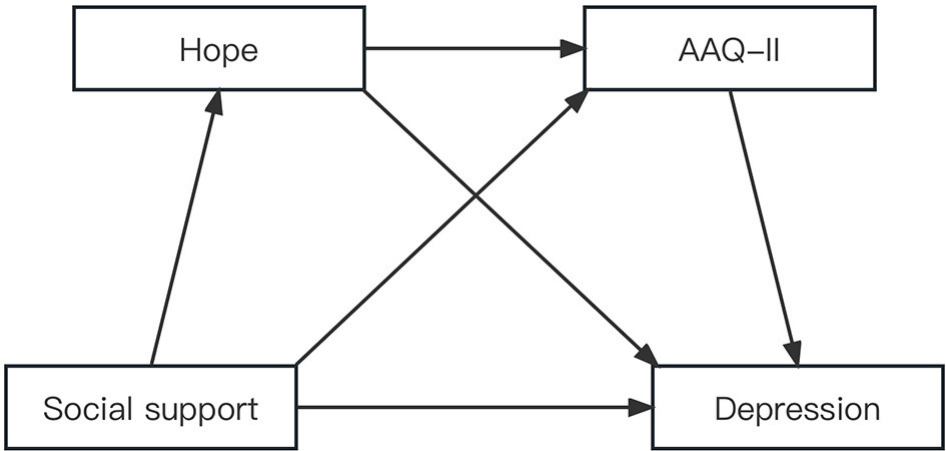
The theroetical models and hypotheses of social support. psychological flexibility, hope, and depression.

In summary, although the relationships among the variables of social support, psychological flexibility, hope, and depression have been examined separately, the role of psychological flexibility and hope in the impact of social support on depression among HIV+ MSM has not yet been thoroughly tested to date. We constructed a hypothetical model to test the relationship between social support and depression and the roles of psychological flexibility and hope among HIV+ MSM. Based on the previous evidence, we put forward four hypotheses: (1) social support might have a direct negative association with depression; (2) psychological flexibility would act as a mediator between social support and depression; (3) hope would act as a mediator between social support and depression; and (4) hope and psychological flexibility would jointly play a chain mediating role in the relationship between social support and depression. The hypothesized theoretical model is illustrated in Figure 1.

## Methods

### Study design and setting

This cross-sectional survey was conducted from June to October 2022. All participants were recruited from the designated HIV/AIDS hospital in Changsha city, Hunan Province of China. Hunan Province is located in south-central China, and the prevalence of HIV is consistent with the national level (38). As of October 2022, the reported cumulative number of surviving PLWHA in Hunan Province exceeded 50,000, and 5,463 were newly reported in 2022, of which 17% were infected through male-to-male sexual transmission, while in Changsha city, this proportion exceeded 50% (39).

### Participants and recruitment

The target population for this study was MSM. The inclusion criteria were as follows: (a) male at birth; (b) self-reported ever having sex with men; (c) 18 years of age or older; (d) diagnosed with HIV infection by Western blot analysis; and (e) living in Hunan Province. Those who (a) had a cognitive impairment that limited their ability to understand and complete the study questionnaires and/or (b) were participating in other psychological interventions were excluded. Convenience sampling was used to recruit participants. The recruitment flier was posted at the hall of a community-based organization (CBO), which was located at the designated hospital. Those who were interested could contact a research assistant for screening. Some potential participants were also referred by the medical social worker from the CBO. This study was approved by the institutional review board of behavioral and nursing research in the Xiangya School of Nursing of Central South University (#E2022121).

### Data collection

Data were collected using an online survey company (http://www.sojump.com), which had signed a contract with the study team to keep the data confidential. Informed consent was obtained from each participant before they started the online survey by scanning a QR code. Only when they clicked the “Informed and Agree” button and signed electronically, they could initiate the survey. IP addresses were used to prevent participants from completing multiple surveys. This survey took approximately 20 min to complete. Participants received 30 RMB (approximately US$5) as compensation for time spent filling out the survey. A total of 300 questionnaires were distributed, of which 290 were valid. Ten questionnaires (3.3%) were excluded due to participants dropping out in the middle of the process, resulting in an efficiency of 96.7%.

### Measures

#### Socio-demographic and disease-related variables

Considering the potential influencing factors of depression (40–45), participants' socio-demographic information, including age, marital status, education level, employment, individual monthly income, and HIV-related information (including years of being diagnosed with HIV, years of antiviral treatment, HIV disclosure status, and drug side effects), was collected.

#### HIV transmission knowledge

A widely used 8-item questionnaire was adopted from “The China AIDS Prevention Supervision and Evaluation Framework Manual” (46). Responses are true or false, with one point for each correct answer. The total score ranges from 0 to 8. A total score of ≥6 indicates good awareness of HIV transmission. In this study, Cronbach's alpha was 0.72.

#### Depression

The Self-rating Depression Scale (SDS) developed by Zung (47) was used. This scale contains 20 items and is scored on a 4-point scale, ranging from “never (1) or occasionally” to “always (4)”. The total score is divided into three levels: low (48–57), moderate (58–67), and high (>72) (68). The higher score indicates a higher level of depressive symptoms. The SDS showed excellent reliability (Cronbach's alpha = 0.89) in China. In the current study, Cronbach's alpha of the scale was 0.82.

#### Social support

Social support was measured using the Social Support Rating Scale (SSRS), developed by Xiao Shuiyuan (69). The scale consists of 10 items, with a total score ranging from 12 to 66. The scale has been widely used in China (69). The higher the total score, the more the social support received. The Chinese SSRS has good validity and reliability (Cronbach's alpha = 0.82). In the current study, Cronbach's alpha of the scale was 0.76.

#### Psychological flexibility

The Acceptance and Action Questionnaire-II (AAQ-II) was used to evaluate the degree of psychological flexibility. Bond et al. (70) revised the AAQ-II scale from 9 items to 7 items, with a total score ranging from 7 to 49. Lower scores indicate lower experiential avoidance and higher psychological flexibility. Jing et al. (71) translated the 7-item scale and applied it to Chinese college students, and it demonstrated good reliability with Cronbach's alpha of 0.88. In the current study, Cronbach's alpha of the scale was 0.94.

#### Hope

The Herth Hope Index (HHI) was used to evaluate each participant's level of hope. The Chinese version of the scale was translated by Haiping and Zian (72). It consists of 12 items, with a total score ranging from 12 to 48. Higher scores indicate higher individual hope. The Chinese version of this scale had good reliability (Cronbach's alpha = 0.85). In the current study, Cronbach's alpha of the scale was 0.77.

### Data analysis

The online questionnaire data were directly exported into an Epidata 3.1 database and cleaned. Statistical analyses were performed using IBM SPSS Statistics 26.0 and PROCESS 3.5.

First, descriptive statistics were calculated. Proportions and frequencies were calculated for the socio-demographic data and disease-related data. The Kolmogorov-Smirnov tests were used to check the normality of the study variables (social support, psychological flexibility, hope, and depression) (48). The mean (M) and standard deviation (SD) were calculated for the study variables if the data met the normal distribution. If not, the median and interquartile range (IQR) of the study variables were calculated. Second, we used Spearman's correlation coefficient to examine the relationships between all study variables among participants. Then, all variables, including socio-demographic and disease-related variables, social support, psychological flexibility, and hope, were entered into the multiple linear regression model to identify the independent correlates of depression among HIV+ MSM. Finally, for each pathway, we used a bias-corrected bootstrap method with 5,000 replications to examine the direct and indirect effects of HIV social support on depression (49). After adjusting covariates that were significant in multivariate regression analysis, the hypothetical single and chain mediation models were examined using PROCESS 3.5 developed by Hayes. Single mediation models of social support for depression through hope and psychological flexibility were examined using the PROCESS model 4 (50). The chain mediation model was carried out using PROCESS model 6 to examine the path between two mediators in sequence and the indirect effects of each mediator independently (49). If the 95% confidence interval did not include zero, the mediating effect was considered statistically significant. Statistical significance was set at *P* < 0.05.

## Results

### Common methods of bias control and testing

Harman's single-factor test was used to examine the common method biases, considering that the data were collected by questionnaire (51). The results showed that the characteristic root of these five factors was >1, and the first factor could explain 20.068%, which was 40% lower than the standard threshold value (51), which indicated that there was no serious common method bias problem in this study.

### General information about the participants

A total of 300 questionnaires were accessed, of which 290 were valid and 10 were discarded. The average age of the participants was 27.71 years (*SD* = 0.417). Most of the participants were unmarried (90.3%) and held a college degree or above (71.4%). Only 9.7% of the participants had an individual monthly income >8,000 RMB (approximately US$1,110). Notably, 64.8% of them had been diagnosed with HIV for more than 6 months, and 50.3% reported 3–6 drug side effects. Less than 1/5 (17.6%) told their partners about their infection status. These data are shown in Table 1.

**Table 1:** General information of the participants (*n* = 290).

**Variables**	***n***	**%**
**Age (years)**		
18–30	194	66.9
31–40	78	26.9
41–51	18	6.2
**Marital status**		
Unmarried	262	90.3
Married	13	4.5
Divorced	15	5.1
**Education level**		
Junior middle school and below	18	6.2
Technical secondary school/high school/vocational school	65	22.4
College or above	207	71.4
**Employment**		
Unemployed	92	31.7
Temporary work	51	17.6
Stationary work	147	50.7
**Individual monthly income (RMB)**		
<3000 (416USD)	84	28.9
3000–5000 (416–694USD)	106	36.6
5001–8000 (694–1110USD)	72	24.8
>8000 (1110USD)	28	9.7
**Years of being diagnosed with HIV (years)**		
≤ 0.5	102	35.2
> 0.5	188	64.8
**Years of antiviral treatment (years)**		
≤ 0.5	195	67.2
> 0.5	95	32.8
**HIV disclosure status**		
Non-disclosure to anyone	94	32.4
Disclosure to families	61	21.0
Disclosure to friends	84	29.0
Disclosure to partners	51	17.6
**Drug side effects (number)**		
<3	83	28.6
3–6	146	50.3
>6	61	21.1

### Description and bivariate correlations of study variables

As shown in Table 2, the mean scores of social support, psychological flexibility, hope, and depression were 25.71 ± 8.665, 28.94 ± 10.969, 34.00 ± 6.676, and 59.00 ± 14.749, respectively. According to these scores, 67.6% (195) had depressive symptoms, and 62.6% (122) of those with depressive symptoms had moderate to severe depressive symptoms.

**Table 2:** Mean, standard deviations, and correlations for study variables (*N* = 290).

**Variable**	**Mean (Median)**	**SD (IQR)**	**(1)**	**(2)**	**(3)**	**(4)**
(1) Social support	25.00	24.71–26.72	1			
(2) Hope	34.00	33.23–34.77	0.570^**^	1		
(3) AAQ-II	29.00	27.67–30.21	−0.381^**^	−0.582^**^	1	
(4) Depression	59.00	14.75	−0.527^**^	−0.729^**^	0.684^**^	1

^**^P <0.01. AAQ-II, Acceptance and Action Questionnaire-II.

Spearman's correlation coefficients between all study variables found that social support had a positive correlation with hope (*r* = 0.570, *P* < 0.01) and negative correlations with AAQ-II (*r* = −0.381, *P* < 0.01) and depression (*r* = −0.527, *P* < 0.01). Hope had a negative correlation with AAQ-II (*r* = −0.582, *P* < 0.01) and depression (*r* = −0.729, *P* < 0.01). Likewise, AAQ-II had a significant positive correlation with depression (*r* = 0.684, *P* < 0.01). Hypothesis 1 was verified.

### Linear regression analysis

In the multiple linear regression model, we used depression as the dependent variable, and socio-demographic variables, disease-related factors, social support, psychological flexibility, and hope as independent variables. At the same time, we tested the collinearity of the independent variables, and the results showed that the tolerance of the independent variables was more than 0.1 and the variance inflation factor (VIF) was <10.0. Therefore, there was no multi-collinearity among independent variables. The results of the regression analysis showed that by controlling for the socio-demographic variables and disease-related factors, social support, psychological flexibility, and hope were significant influencing factors of depression for HIV+ MSM (Table 3). In addition to the study variables, age, individual monthly income, years of being diagnosed with HIV, HIV disclosure status, drug side effects, and HIV transmission knowledge score were also influential factors for depression in HIV+ MSM.

**Table 3:** Linear regression of factors associated with depression.

**Factor**	**Unstandardized coefficients**		**Standardized coefficients**	***t***	***P***
	***B***	***SE***	β		
Constant	97.286	6.136		15.854	0.000
**Age (ref: 18–30, years)**					
31–40	0.293	1.140	0.009	0.257	0.797
41–51	−4.795	2.387	−0.079	−2.009	0.046
**Marital status (ref: Unmarried)**					
Married	1.846	2.486	0.026	0.743	0.458
Divorced	−1.055	2.540	−0.016	−0.415	0.678
**Education level (ref: Junior middle school and below)**					
Technical secondary school/high school/vocational school	2.860	2.043	0.047	1.400	0.163
College and above	0.502	1.153	0.014	0.435	0.664
**Working status (ref: Unemployed)**					
Temporary work	0.851	1.640	0.022	0.519	0.604
Stationary work	2.735	1.613	0.093	1.695	0.091
**Individual monthly income [ref: <3000 (416USD), RMB]**					
3000–5000 (416–694USD)	−0.675	1.603	−0.022	−0.421	0.674
5001–8000 (694–1110USD)	−3.133	1.829	−0.092	−1.713	0.088
>8000 (1110USD)	−5.605	2.164	−0.112	−2.590	0.010
**Years of being diagnosed with HIV (ref:** ≤ **0.5, years)**					
> 0.5	−2.676	1.376	−0.087	−1.945	0.043
**Years of antiviral treatment (ref:** ≤ **0.5, years)**					
> 0.5	−1.942	1.352	−0.062	−1.436	0.152
**HIV disclosure status (ref: Non-disclosure to anyone)**					
Disclosure to families	0.498	1.311	0.014	0.380	0.705
Disclosure to friends	−0.990	1.172	−0.031	−0.844	0.399
Disclosure to partners	−7.665	1.632	−0.183	−4.698	0.000
**Drug side effects (ref: <3, number)**					
3–6	2.526	1.166	0.086	2.167	0.031
>6	7.598	1.485	0.210	5.118	0.000
HIV transmission knowledge	−1.756	0.620	−0.091	−2.830	0.005
Social support	0.346	0.054	0.258	6.420	0.000
AAQ-II	−0.874	0.092	−0.396	−9.451	0.000
Hope	−0.169	0.063	−0.099	−2.690	0.008

R^2^ = 0.871, F = 40.299, p <0.000.

### The single mediating effect of hope and psychological flexibility on the relationship between social support and depression

**Figure 2:**
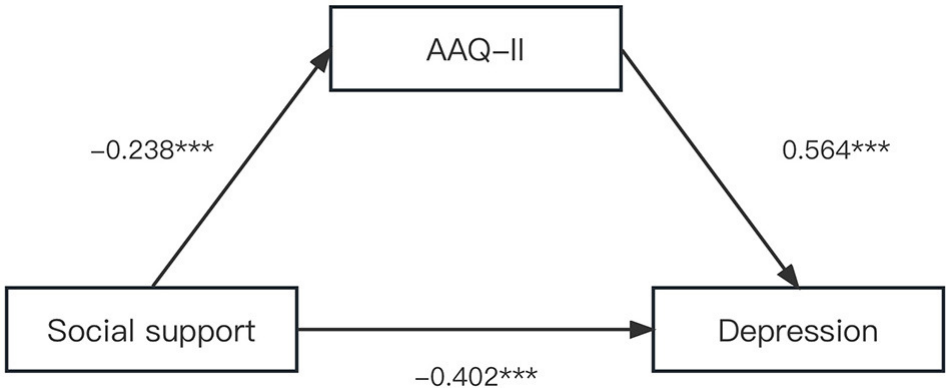
The single mediation role of psychological flexibility in the relationship between social support and depression. The solid line indicated significant path coefficients. ^***^p < 0.001.

We examined the single mediation effect of psychological flexibility and hope on the relationship between social support and depression after controlling HIV disclosure and drug side effects, respectively. As shown in Figure 2, in the model with psychological flexibility as the single mediator, social support was positively associated with psychological flexibility (β = −0.238, *P* < 0.001), and psychological flexibility was negatively associated with depression (β = 0.564, *P* < 0.001). A significant indirect effect of social support on depression via psychological flexibility was found, and the mediating effect value was −0.134 [Bootstrap 95% CI: −0.235, −0.059]. Therefore, psychological flexibility played a partial mediating role between social support and depression. Hypothesis 2 was verified.

**Figure 3:**
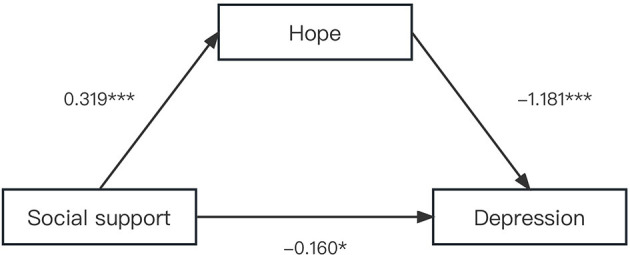
The single mediation role of hope in relationship between social support and depression. The solid line indicated significant path coefficients. ^***^p < 0.001 and ^*^p < 0.05.

As shown in Figure 3, in the model with hope as the mediator, social support was positively associated with hope (β = 0.319, *P* < 0.001), and hope was negatively associated with depression (β = –1.181, *P* < 0.001). A significant indirect effect of social support on depression via hope was found. The mediating effect of hope was −0.376 [Bootstrap 95% CI: −0.503, −0.266]. Hypothesis 3 was supported.

### The chain mediating effect of hope and psychological flexibility on the relationship between social support and depression

**Figure 4:**
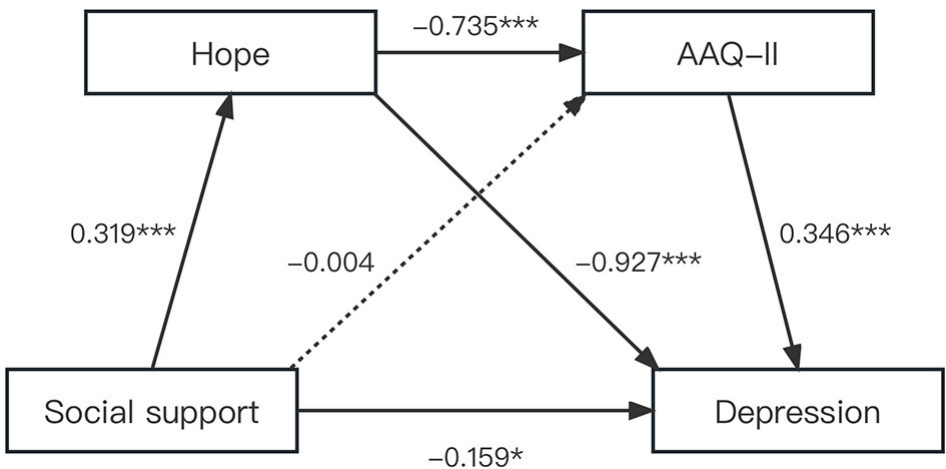
The chain mediation role of psychological flexibility in the relationship between social support and depression. The solid and dashed line indicated significant and non-significant path coefficients. ^***^p < 0.001 and ^*^p < 0.05.

Regression analysis was carried out on the chain intermediary effect model (52). Table 4 displays the coefficients and significance of each path in the chain mediation model. We found that social support was negatively associated with depression. The total effect was −0.537 [Bootstrap 95% CI: −0.760, −0.367], and the direct effect was −0.159 [Bootstrap 95% CI: −0.298, −0.020], which meant that the higher the level of social support, the lower the depressive symptoms were among HIV+ MSM. Therefore, hypothesis 1 was supported. Table 4 confirmed that social support had a direct and significant positive association with hope (β = 0.319, *P* < 0.001), while it had no significant association with psychological flexibility (β = −0.004, *P* = 0.966). Hope was negatively associated with depression (β = −0.927, *P* < 0.001) and positively associated with psychological flexibility (β = −0.735, *P* < 0.001). Psychological flexibility had a significant negative association with depression (β = 6.093, *P* < 0.001). Meanwhile, social support was still negatively associated with depression (β = −0.159, *P* < 0.05; Figure 4). It could be concluded that psychological flexibility and hope played an intermediary role between social support and depression. Hypotheses 1 and 3 were supported by data.

**Table 4:** Analysis of regression relationship of variables.

**Predictor variable**	**R2**	**F**	**β**	**t**	**p**	**LLCI**	**ULCI**
**Equation 1 outcome variable: hope**							
Social support	0.600	28.028	0.319	7.834	0.000	0.239	0.399
**Equation 2 outcome variable: AAQ-II**							
Hope	0.624	41.674	−0.735	−6.357	0.000	−0.962	−0.507
Social support			−0.004	−0.043	0.966	−0.174	0.167
**Equation 3 outcome variable: depression**							
Hope	0.853	91.545	−0.927	−8.810	0.000	−1.134	−0.720
AAQ-II			0.346	6.093	0.000	0.234	0.458
Social support			−0.159	−2.254	0.025	−0.298	−0.020

Table 5 shows the results of the chain mediation analyses and the effect size of the intermediary pathway, which indicated that the total indirect effect was −0.378 [Bootstrap 95% CI: −0.510, −0.273], while the direct effect was −0.159 [Bootstrap 95% CI: −0.298, −0.020], suggesting that the indirect effect was stronger than the direct effect. Specifically, the total indirect effect of the relationship between social support and depression included two pathways, and all specific mediating effects and chain mediating effects were significant. The indirect mediating effect of hope was −0.295 [Bootstrap 95% CI: −0.408, −0.203]. The bootstrap's 95% CI of the path did not contain zero, indicating that this indirect path was statistically significant. Importantly, the indirect effect of chain mediation from social support to depression via hope and psychological flexibility was significant, and the effect value was −0.082 [Bootstrap 95% CI: −0.129, −0.048]. The total indirect effect of psychological flexibility and hope accounted for 70.39% in the chain model, while the direct effect accounted for only 29.61% (Figure 4). Therefore, hypothesis 4 was supported.

**Table 5:** Total, direct, and indirect effect of social support on depression though psychological flexibility and hope.

**Effect**	**Estimate**	**Boot SE**	**Bootstrap 95% CI**		**Effect size (%)**
			**Low**	**High**	
Total effects	−0.537	0.086	−0.706	−0.367	100.00
Direct effects	−0.159	0.071	−0.298	−0.020	29.610
Total indirect effects	−0.378	0.060	−0.510	−0.273	70.390
X → M1 → Y	−0.295	0.053	−0.408	−0.203	54.930
X → M2 → Y	−0.001	0.030	−0.062	0.054	/
X → M1 → M2 → Y	−0.082	0.020	−0.129	−0.048	15.270

X, social support; Y, depression; M1, hope; M2, psychological flexibility.

## Discussion

This study explored the intermediary mechanism between social support and depression among Chinese HIV+ MSM based on the stress-processing model. The results supported the hypotheses and indicated that psychological flexibility and hope played a chain-medicating role in the association between social support and depression. In addition, the chain mediation analyses suggested a significant serial effect between psychological flexibility and hope.

In this study, the prevalence of depression among Chinese HIV+ MSM was 67.6% (196/290), which was much higher than that reported globally (43%) (7) and in other settings such as Vancouver, Canada (39.3%) (53) and the United States (40.8%) (54). Compared to the United States and European countries (55–57), China exhibits higher judgmental attitudes toward homosexuality due to traditional social norms and the Confucian philosophy that emphasizes heterosexual marriage and having sons to pass down the family name (58). Thus, the majority of Chinese people do not accept homosexuals and hold negative attitudes such as intolerance and avoidance toward MSM (59). When MSM get infected with HIV, they experience much more stress due to the HIV-related stigma, which adds to the homosexual stigma. HIV-related stigma in China is also higher than that in the West and some African countries (60, 61). As HIV infection is often considered to be associated with moral degradation or promiscuity, which leads to social discrimination and exclusion (40), Chinese PLWHA face more social condemnation, discrimination, and stigma (13, 59, 62, 63). Chinese PLWHA also experience higher stigma compared to some African countries, where HIV prevalence is very high, such as Kenya (22.02%), Zambia (23.38%), and Lesotho (25.48%) (64), and the public is not afraid of HIV as in China. The literature has confirmed that the dual stigma could lead to an increased prevalence of depression for HIV+ MSM. A meta-analysis found a 43% co-prevalence of depression and HIV infection among MSM, and HIV+ MSM were more likely to be depressed compared to HIV-negative MSM (65). In addition, evidence indicated that stigma and discrimination against sexual orientation and HIV infection were major sources of stress for HIV+ MSM (14, 66, 67). Therefore, HIV and depression among HIV+ MSM in China are syndemic, creating a vicious cycle in the HIV epidemic (20).

Moreover, as an isolated and marginalized group, HIV+ MSM perceived lower social support compared with that reported in previous studies (73, 74). In addition to the high dual stigma, which reduced the available social support resources, the concealment of their sexual orientation and HIV status also limited their access to those supporting resources. According to a recent meta-analysis, the disclosure rates for HIV+ MSM to their family, intimate friends, spouses, and regular sexual partners were low, at 43.42, 47.9, 56.8, and 43.2%, respectively (40). Chinese MSM usually face the dilemma of being exposed as gay men once informed of their HIV status, and they also fear hurting family feelings and wish to avoid embarrassment (75). Furthermore, the disclosure of HIV status causes familial exclusion, engenders a sense of social isolation, and gives rise to serious consequences such as impaired social roles and relationships, leading HIV+ MSM to experience less access to social support (76). Thus, medical staff should leverage proximity to facilitate early disclosure to help ensure HIV+ MSM receive adequate psychological care from their loved ones.

Consistent with hypothesis 3, this study found that hope partially mediated the relationship between social support and depression. Although the pathways have not been fully illuminated, some studies provided indirect support for them. For example, Masquillier et al. (15) confirmed that family functioning was positively associated with hope among PLWHA, and a supportive family could provide spiritual support to patients. The more patients felt being cared for by family members, the higher their level of hope. Furthermore, hope has a close association with anxiety and depression. Substantial evidence indicates that PLWHA with higher levels of hope have less depression and anxiety and a greater ability to cope with difficulties (31, 33, 34). The present study further supports these findings and suggests that social support, serving as an intrinsic resource, could strengthen hope, increase confidence, and ultimately reduce psychological distress, including depression.

This study found that psychological flexibility was not a mediator between social support and depression, which might be related to our sample characteristics and study methodology. Nevertheless, we found that social support did play an important role in reducing depression in HIV+ MSM, which was consistent with previous findings (13, 26). Furthermore, our most noteworthy finding was that social support had an effect on depression through the chain mediation of hope and psychological flexibility, which means that, with more social support, HIV+ MSM will first feel more hope and then increase psychological flexibility, which ultimately alleviates their depressive symptoms. These findings support our hypothesis and extend the existing evidence indicating that hope is a core dimension of recovery from mental disorders and negative coping styles (15). Nyoni et al. (77) conducted a study among 346 PLWHA in sub-Saharan Africa and suggested that perceived social support, especially family support, could enhance a sense of hope, establish a positive attitude, help them adapt to external changes, and then find appropriate self-regulation mechanisms, thereby reducing depressive symptoms. In addition, our study demonstrated a positive association between hope and psychological flexibility. Evidence confirmed that patients with higher levels of hope might have more conviction and motivation to face potentially traumatic events and thus have higher levels of psychological flexibility and resilience in the face of stress and difficulties (35). Similarly, Landstra et al. (26) also confirmed that some resilience-promoting resources, such as optimism, hope, and self-efficacy, could enhance psychological flexibility and ultimately reduce the incidence of depression. In addition, the stress process model (12) could explain this chain-mediating effect. The model suggests that an individual's perception of coping resources (e.g., social support) affects his or her hope and confidence in regulating stress, which in turn affects flexibility and adaptability in the face of stress and difficulties (e.g., psychological flexibility) and ultimately determines the extent to which mental disorders increase or decrease (78). Therefore, incorporating the concepts of hope and psychological flexibility into interventions can provide better mental health support for HIV+ MSM and improve their quality of life and wellbeing.

Inevitably, this study had several limitations that should be noted. First, the participants were only from one city in China, and thus the results might not be generalizable to HIV+ MSM populations in other geographic regions. Second, the cross-sectional and self-reported nature of the study might cause recall bias. In addition, this study only controlled for demographic and some disease-related variables; other potential influencing variables were not collected, such as coping strategies (79), which might affect the stability of the chain mediating model.

Despite these limitations, this study revealed the mediating role of psychological flexibility and hope between social support and depression, which has several implications for future research and practice. First, this study illustrates the need for interventions to improve the mental health of HIV+ MSM. Positive psychological interventions can play an important role in alleviating depressive symptoms and enhancing wellbeing in PLWHA (4, 20). Therefore, researchers need to adopt a positive perspective in addressing the mental health disorders of patients and explore new pathways for their psychological care. Future intervention studies can specifically target the psychological flexibility and hope of HIV+ MSM to improve their mental health and prevent HIV transmission. Second, a more conducive social environment should be created. Promoting social acceptance for MSM (10), reducing HIV-related discrimination and stigma, and improving social support (80) can provide emotional support to HIV+ MSM and reduce negative emotions (10, 13, 80). This underscores the importance of considering not only individual-level interventions but also social factors that affect the mental health of HIV+ MSM. In addition, public health policymakers should strive for clear policies and practices to promote social equity and medical convenience for MSM (76). For instance, expanding the PrEP transport network and HIV self-testing services can enhance the sustainability of PrEP among MSM (81, 82). Research conducted by Schanll et al. (82) highlights the high risk of HIV infection among young MSM (YMSM), underscoring the potential utility of HIV self-testing as a tool to ensure timely detection of infection and initiation of antiretroviral therapy. Finally, some current interventions focus only on social support (83), hope (15), or psychological flexibility (25) as ways to improve the mental health of PLWHA, but our study suggests that future interventions should combine the efforts to stimulate social support by increasing the level of hope and enhancing psychological flexibility. Integrated interventions are urgently needed to address structural barriers to accessing HIV prevention and treatment (84), thereby achieving the UNAIDS' 95-95-95 targets for ending AIDS by 2030 (85).

## Conclusion

This study clarified the mediating pathway between social support and depression among Chinese HIV+ MSM. These findings indicated that hope and psychological flexibility play a chain-mediating role in this relationship and that hope also plays a single-mediating role. Given the mediating role of psychological flexibility and hope on depression among HIV+ MSM, interventions related to psychological flexibility and hope should be designed to enhance mental health among HIV+ MSM in China and other global settings.

## Data availability statement

The original contributions presented in the study are included in the article/supplementary material, further inquiries can be directed to the corresponding authors.

## Ethics statement

The studies involving humans were approved by the Institutional Review Board of Behavioral and Nursing Research in the Xiangya School of Nursing of Central South University. The studies were conducted in accordance with the local legislation and institutional requirements. The participants provided their written informed consent to participate in this study.

## Author contributions

RW: Data curation, Formal analysis, Investigation, Methodology, Project administration, Supervision, Validation, Writing—original draft, Writing—review & editing, Software. FZ: Writing—review & editing. GC: Writing—review & editing. LG: Writing—review & editing. YS: Writing—review & editing. CZ: Writing—review & editing. MY: Writing—review & editing. WP: Writing—review & editing. XL: Writing—review & editing, Resources, Supervision.
